# GWAS and transcriptional analysis prioritize *ITPR1* and *CNTN4* for a serum uric acid 3p26 QTL in Mexican Americans

**DOI:** 10.1186/s12864-016-2594-5

**Published:** 2016-04-02

**Authors:** Geetha Chittoor, Jack W. Kent, Marcio Almeida, Sobha Puppala, Vidya S. Farook, Shelley A. Cole, Karin Haack, Harald H. H. Göring, Jean W. MacCluer, Joanne E. Curran, Melanie A. Carless, Matthew P. Johnson, Eric K. Moses, Laura Almasy, Michael C. Mahaney, Donna M. Lehman, Ravindranath Duggirala, Anthony G. Comuzzie, John Blangero, Venkata Saroja Voruganti

**Affiliations:** Department of Nutrition, University of North Carolina at Chapel Hill, Chapel Hill, NC USA; UNC Nutrition Research Institute, University of North Carolina at Chapel Hill, 500 Laureate Way, Kannapolis, NC 28081 USA; Department of Genetics, Texas Biomedical Research Institute, San Antonio, TX USA; South Texas Diabetes and Obesity Institute, School of Medicine, University of Texas Rio Grande Valley, Brownsville, TX USA; Centre for Genetic Epidemiology and Biostatistics, The University of Western Australia, Perth, WA Australia; Department of Medicine, University of Texas Health Science Center at San Antonio, San Antonio, TX USA

**Keywords:** Joint linkage/association approach, *CNTN4*, *ITPR1*, Family-based study

## Abstract

**Background:**

The variation in serum uric acid concentrations is under significant genetic influence. Elevated SUA concentrations have been linked to increased risk for gout, kidney stones, chronic kidney disease, and cardiovascular disease whereas reduced serum uric acid concentrations have been linked to multiple sclerosis, Parkinson’s disease and Alzheimer’s disease. Previously, we identified a novel locus on chromosome 3p26 affecting serum uric acid concentrations in Mexican Americans from San Antonio Family Heart Study. As a follow up, we examined genome-wide single nucleotide polymorphism data in an extended cohort of 1281 Mexican Americans from multigenerational families of the San Antonio Family Heart Study and the San Antonio Family Diabetes/Gallbladder Study. We used a linear regression-based joint linkage/association test under an additive model of allelic effect, while accounting for non-independence among family members via a kinship variance component.

**Results:**

Univariate genetic analysis indicated serum uric acid concentrations to be significant heritable (*h*^2^ = 0.50 ± 0.05, *p* < 4 × 10^−35^), and linkage analysis of serum uric acid concentrations confirmed our previous finding of a novel locus on 3p26 (LOD = 4.9, *p* < 1 × 10^−5^) in the extended sample. Additionally, we observed strong association of serum uric acid concentrations with variants in following candidate genes in the 3p26 region; inositol 1,4,5-trisphosphate receptor, type 1 (*ITPR1*), contactin 4 (*CNTN4*), decapping mRNA 1A (*DCP1A*); transglutaminase 4 (*TGM4*) and rho guanine nucleotide exchange factor (GEF) 26 (*ARHGEF26*) [*p* < 3 × 10^−7^; minor allele frequencies ranged between 0.003 and 0.42] and evidence of *cis*-regulation for ITPR1 transcripts.

**Conclusion:**

Our results confirm the importance of the chromosome 3p26 locus and genetic variants in this region in the regulation of serum uric acid concentrations.

## Background

The end product of purine metabolism in humans and higher order primates is uric acid, which cannot be further broken down because of lack of uricase [1]. Elevated serum uric acid (SUA) levels or hyperuricemia, a metabolic risk factor for gout and cardio-renal diseases, has been increasing in prevalence worldwide [2–8]. As other cardiovascular and renal disease risk factors, hyperuricemia also has a strong genetic basis [9–11]. SUA is a complex trait, and its pattern of inheritance suggests that several genes may influence it. Numerous genome-wide and candidate gene studies have found various genes, mostly uric acid transporters, to be significantly associated with SUA in several populations, such as solute carrier protein 2 family, member 9 (*SLC2A9*) [12–17], solute carrier protein 22 family, member 11 (*SLC22A11*), solute carrier protein 17 family members 1 and 3 (*SLC17A1, SLC17A3*), solute carrier protein 16 family member 9 (*SLC16A9*) and ATP-binding cassette, subfamily G. member 2 (*ABCG2*) [18–21].

In a previous linkage study in 632 Mexican Americans of the San Antonio Family Heart Study, we found strong evidence of linkage for SUA concentrations on 3p26 (LOD = 4.2) [9] and suggestive evidence of association with the positional candidate gene contactin 4 (*CNTN4*) [22]. Other candidate genes in this region are inositol 1,4,5-trisphosphate receptor, type 1 (*ITPR1*), decapping mRNA 1A (*DCP1A*); transglutaminase 4 (TGM4) and rho guanine nucleotide exchange factor (GEF) 26 (*ARHGEF26*). The one LOD-confidence interval on 3p26 falls within the candidate region for 3p deletion syndrome whose features include developmental delays and mental retardation [21]. In particular, disruption of *CNTN4* and *ITPR1* seem to contribute to the 3p deletion syndrome phenotype and may have a causal relationship [22, 23]. SUA is also known as a biomarker for neurodegenerative diseases such as dementia, stroke, Parkinson’s disease and multiple sclerosis [24–28].

Given that we previously identified 3p26 quantitative trait locus (QTL) regulating SUA concentrations, a detailed understanding of the genetic architecture of all candidate genes/variants within this 3p region and its association with SUA is crucial. The aim of this study was to assess the association of variants in the chromosome 3p region in an expanded cohort of 1281 Mexican Americans from the San Antonio Family Heart Study (SAFHS) and the San Antonio Family Diabetes/Gallbladder study (SAFDGS).

## Results

The mean ± SD of age and SUA levels of participating individuals (*n* = 1281) were 46.64 ± 15.8 years and 5.80 ± 1.6 mg/dl, respectively, with men having higher levels of SUA than women (6.68 ± 1.6 vs. 5.28 ± 1.4) (Table 1). Significant heritability was detected for SUA levels (*h*^2^ = 0.50 ± 0.05, *p* = 3.2 × 10^−35^) with age, sex, and interaction between age and sex as covariates.

**Table 1:** Descriptive characteristics and heritability estimates of serum uric acid (mg/dl)

Variable	Males		Females		Total Population				
	*N*	Mean ± SD	*N*	Mean ± SD	*N*	Mean ± SD	*h* ^*2*^ ± SE	*p*-value	Sig. Covariates
Age (years)	471	46.01 ± 16.21	810	47.00 ± 15.53	1281	46.64 ± 15.78	–	–	–
Serum uric acid (mg/dl)^a^	471	6.68 ± 1.64	810	5.28 ± 1.35	1281	5.80 ± 1.61	0.50 ± 0.05	3.2 × 10^−35^	Age, Sex, Age*Sex

^a^Rank-inverse-normal transformed data used for genetic analyses

### Genome-wide joint linkage/Association analysis

**Fig. 1:**
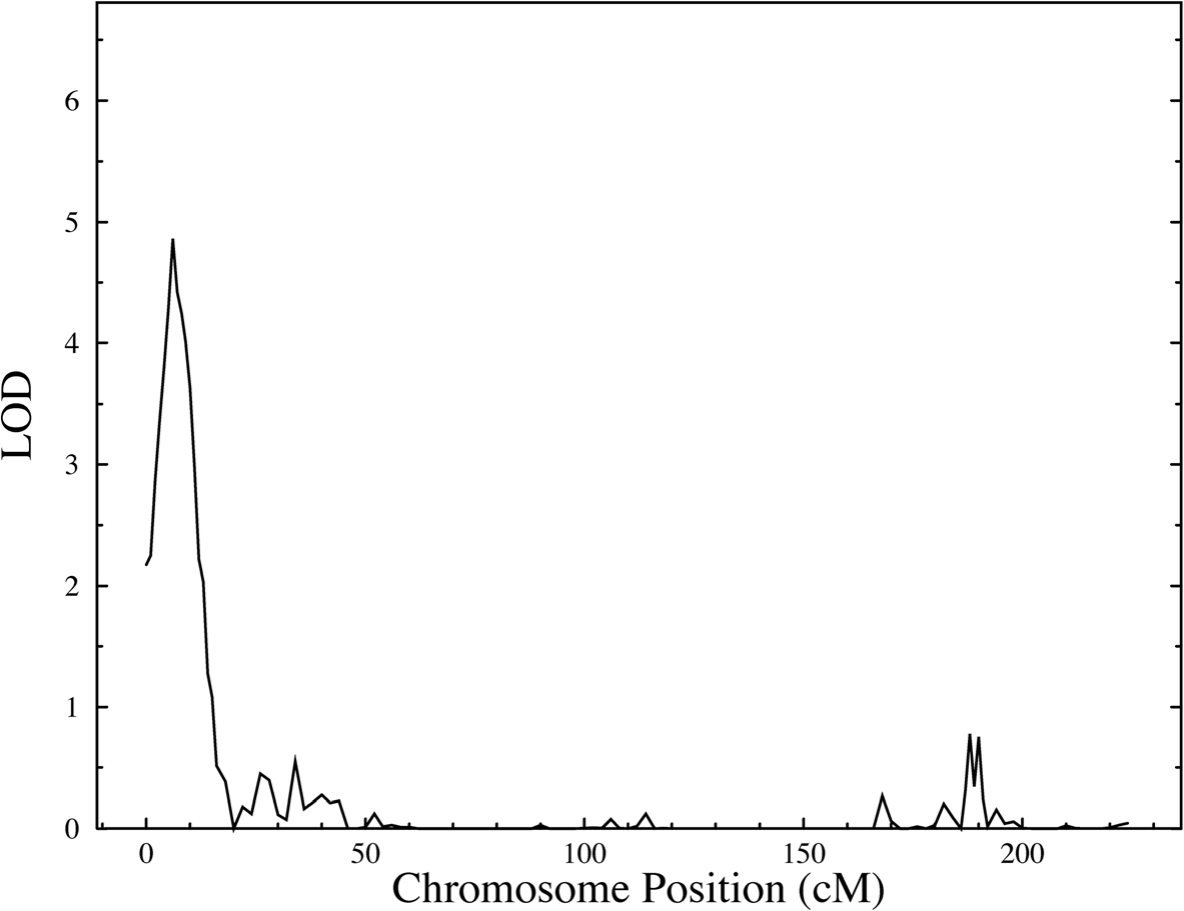
Joint linkage association analysis of serum uric acid on chromosome 3p26 showing a strong signal (LOD = 4.9) in Mexican Americans

**Fig. 2:**
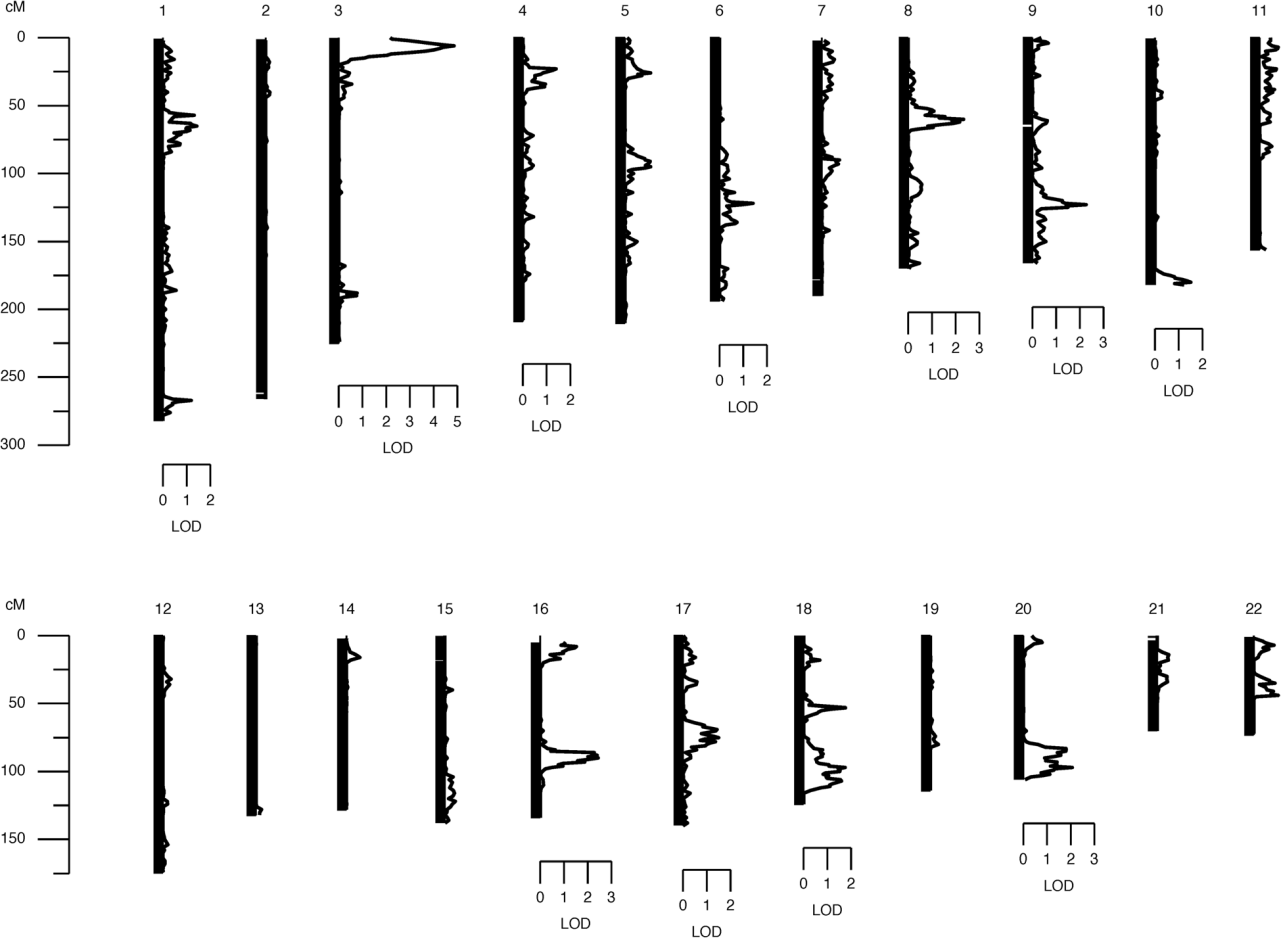
Chromosomal regions linked to serum uric acid in a genome-wide scan with multiopoint LOD scores ≥ 1.2

Prior to genetic analysis, SUA was rank-inverse-normal transformed and regressed on age, sex, and interaction between age and sex. Joint linkage analysis (JLA) results confirmed our previous findings [9] with the strongest evidence for linkage of SUA on 3p26 (LOD = 4.9, *p* = 1 × 10^−6^) (Fig. 1). We observed strong association between SUA concentrations and SNPs in candidate genes in the one-LOD confidence interval of 3p26; inositol 1,4,5-trisphosphate receptor, type 1 (*ITPR1*), rs11916691 (A): decapping mRNA 1A (*DCP1A*), rs1395388 (G): transglutaminase 4 (*TGM4*)*,* and contactin 4 (*CNTN4*) (Table 2). The minor alleles of eight of these SNPs are associated with lower levels of SUA concentrations. The minor allele frequencies (MAFs) (range 0.3 to 43 %), and results of association analysis of these SNPs with SUA are given in Table 2. Genotypic-specific mean values of SUA for significant and suggestive associated SNPs are given in Table 3. In addition, the genome-wide linkage screen localized suggestive evidence of linkage of SUA with QTLs on chromosomes 8, 9, 16, and 20, respectively (LOD ≥ 2.0) (Fig. 2).

**Table 2:** Joint linkage-association analysis of serum uric acid (mg/dl) on chromosome 3

SNP^a^	Gene	Coordinates NCBI36 (bp)	JLA^b^ (*p*-value)	MGA^c^ (*p*-value)	Minor allele/frequency
rs17040820	*ITPR1* ^*d*^	4531694	1.3 × 10^−10^	9.4 × 10^−10^	T/0.003
rs7640752	*Intergenic*	114123136	8.6 × 10^−10^	1.9 × 10^−10^	A/0.005
rs11916691	*DCP1A* ^*e*^	53350244	9.2 × 10^−10^	2.0 × 10^−10^	A/0.005
rs1395388	*TGM4* ^*f*^	44923678	1.8 × 10^−8^	4.2 × 10^−9^	G/0.05
rs449361	*ARHGEF26* ^*g*^	155405100	2.8 × 10^−8^	6.6 × 10^−9^	T/0.05
rs1014805	*CNTN4* ^*h*^	2310471	7.7 × 10^−8^	9.1 × 10^−4^	C/0.33
rs2535632	*ITIH4* ^*i*^	52839315	1.8 × 10^−7^	4.3 × 10^−8^	T/0.03
rs9854606	*CNTN4*	2380442	1.9 × 10^−7^	2.9 × 10^−3^	T/0.004
rs1685456	*CNTN4*	2321860	2.1 × 10^−7^	1.8 × 10^−3^	C/0.43
rs1685447	*CNTN4*	2313137	2.1 × 10^−7^	3.4 × 10^−3^	A/0.27
rs1178487	*CNTN4*	2315743	2.2 × 10^−7^	3.0 × 10^−3^	T/0.41
rs17013501	*CNTN4*	2396201	2.5 × 10^−7^	5.0 × 10^−3^	T/0.22
rs6808240	*CNTN4*	2397321	3.3 × 10^−7^	8.8 × 10^−3^	C/0.27
rs1178492	*CNTN4*	2318040	3.7 × 10^−7^	7.7 × 10^−3^	T/0.27
rs1502582	*CNTN4*	2326831	3.7 × 10^−7^	6.7 × 10^−3^	T/0.24
rs1720201	*CNTN4*	2313231	4.4 × 10^−7^	1.1 × 10^−2^	G/0.27

^a^SNP: Single Nucleotide Polymorphism; ^b^JLA: Joint Linkage Association Analysis; ^c^MGA: Measured Genotype Analysis; ^d^ *ITPR1*: inositol 1,4,5-trisphosphate receptor, type 1; ^e^ *DCP1A*: decapping mRNA 1A; ^f^ *TGM4*: transglutaminase 4; ^g^ *ARHGEF26*: Rho guanine nucleotide exchange factor (GEF) 26; ^h^ *CNTN4*: Contactin 4; ^i^ *ITIH4*: inter-alpha-trypsin inhibitor heavy chain family, member 4

**Table 3:** Genotype-specific phenotype means of serum uric acid (mg/dl) concentrations for significant and suggestive associations

SNP^a^	Genotype-specific phenotype means [Mean (SD)]			Effect size^b^ (%)
	Minor/minor	Minor/major	Major/major	
rs17040820	–	7.15 (1.2)	5.56 (1.5)	5.2
rs7640752	–	5.13 (1.8)	5.57 (1.5)	5.4
rs11916691	–	6.63 (0.8)	5.56 (1.5)	5.4
rs1395388	–	5.74 (1.6)	5.55 (1.5)	4.7
rs449361	4.3 (0.3)	5.76 (1.6)	5.56 (1.5)	4.7
rs1014805	5.82 (1.6)	5.89 (1.6)	5.70 (1.6)	0.6
rs2535632	–	5.27 (1.3)	5.58 (1.5)	4.4
rs9854606	–	6.77 (1.8)	5.53 (1.5)	1.1
rs1685456	5.93 (1.7)	5.83 (1.5)	5.66 (1.6)	0.7
rs1685447	5.30 (1.3)	5.69 (1.5)	5.52 (1.6)	0.8
rs1178487	5.93 (1.6)	5.82 (1.6)	5.70 (1.7)	0.6
rs17013501	5.69 (1.5)	5.65 (1.5)	5.89 (1.7)	0.5
rs6808240	6.00 (1.7)	5.93 (1.7)	5.66 (1.5)	0.4
rs1178492	5.70 (1.7)	5.90 (1.6)	5.73 (1.6)	0.5
rs1502582	5.71 (1.8)	5.91 (1.5)	5.74 (1.6)	0.4
rs1720201	5.77 (1.6)	5.88 (1.6)	5.74 (1.6)	0.4
rs1562692	–	4.92 (1.2)	5.61 (1.5)	3.7

^a^SNP: Single Nucleotide Polymorphism; ^b^Effect size: Proportion of the residual phenotypic variance that is explained by the minor allele of the SNP

### Genetic analysis of expression levels of genes in the chromosome 3p region

As a next step, we performed genetic analysis to estimate heritabilities of gene expression of candidate genes in chromosome 3p26 region. Significant heritabilities were observed for *ITPR1* (*h*^2^ = 0.21 ± 0.5, *p* = 3 × 10^−7^). Previously, transcriptomic analysis in SAFHS [29] identified several *cis*-regulated transcripts including *ITPR1*. Genome-wide association analysis provided evidence of association between *ITPR1* expression and *ITPR1* SNPs, and sodium channel, voltage gated, type VIII alpha subunit (*SCN8A*) genes (p between 10^−5^ and 10^−7^). In addition, *ITPR1* showed suggestive associations with SNPs in intergenic regions in chromosomes 1, 2, 10 and 12 (Table 4).

**Table 4:** Genome-wide association of *ITPR1* transcript levels

SNP^a^	Gene^b^	Chr	Coordinates NCBI36 (bp)	MGA^c^ (*p*-value)	JLA^d^ (*p*-value)	Effect size^e^ (%)	Minor allele	MAF^f^
rs877850	*Intergenic*	1	216328255	2.35 × 10^−7^	9.05 × 10^−7^	2.61	G	0.12
rs9311419	*ITPR1*	3	4855671	9.12 × 10^−7^	3.37 × 10^−6^	2.58	T	0.14
rs4685832	*ITPR1*	3	4859817	1.19 × 10^−6^	4.37 × 10^−6^	2.50	G	0.16
rs12581731	*SCN8A*	12	50311004	2.54 × 10^−6^	8.89 × 10^−6^	1.97	A	0.12
rs3805034	*ITPR1*	3	4855266	4.22 × 10^−6^	1.48 × 10^−6^	2.31	A	0.12
rs3805035	*ITPR1*	3	4855300	4.94 × 10^−6^	1.72 × 10^−6^	2.30	T	0.13
rs10886848	*Intergenic*	10	122831260	1.41 × 10^−5^	3.44 × 10^−5^	1.73	A	0.46
rs10170245	*LOC105373893*	2	220897407	1.61 × 10^−5^	5.36 × 10^−5^	1.67	A	0.09
rs4561600	*Intergenic*	2	142915296	1.71 × 10^−5^	5.70 × 10^−5^	1.58	G	0.17
rs4553758	*Intergenic*	2	142914017	1.83 × 10^−5^	6.07 × 10^−5^	1.50	A	0.21

^a^SNP: Single nucleotide polymorphism; ^b^ *ITPR1*: inositol 1,4,5-trisphosphate receptor, type 1; *SCN8A*: sodium channel, voltage gated, type VIII alpha subunit; ^c^MGA: Measured Genotype Analysis; ^d^JLA: Joint Linkage Association Analysis; ^e^Effect size: Proportion of the residual phenotypic variance that is explained by the minor allele of the SNP; ^f^MAF: Minor Allele Frequency

## Discussion

Our results demonstrate the importance of chromosome 3p26 genetic variants in the regulation of SUA concentrations in Mexican Americans. We identified a QTL with significant evidence of linkage on chromosome 3 (LOD = 4.9) for SUA in an expanded cohort, confirming our previous linkage of a novel QTL on chromosome 3p26 affecting SUA and better reflecting pedigree-specific effects. This region has been reported to harbor positional candidate genes with potential relevance to cardiovascular disease, hypertension, obesity, and metabolic syndrome [2–5]. *CNTN4*, a candidate gene in the linkage region of 3p26, is a member of the contactin subgroup of cell adhesion molecules of the immunoglobulin (Ig) superfamily and plays an important role in maintenance and plasticity of functional neuronal networks and central nervous system (CNS) development [23]. The variants in this gene are associated with developmental delays and mental retardation and may be relevant to autism-related spectrum disorders [30–32]. Disruption of *CNTN4* is also thought to cause cognitive defects [33]. Our study showed strong association of SUA concentrations with *CNTN4* SNPs. Specifically, one *CNTN4* variant, rs9854606 is notable with a minor allele frequency (T) of 0.4 %. Although, SUA has not been associated with autism or related disorders, it has been considered a biomarker for neurological disorders such as Parkinson’s disease [34], multiple sclerosis [35] and Alzheimer’s disease [36, 37] and cognitive defects [38, 39].

Other genes in our QTL region, *ITPR1*, *DCP1A* and *TGM4*, do not seem to have functional relevance to SUA concentrations. However, all of these genes are located in or border the ~4.5 Mb region which is associated with a syndrome known as 3p deletion syndrome. Individuals with 3p deletion syndrome have a rare genetic disorder characterized by developmental delay, growth retardation and dysmorphic features [22]. *ITPR1* encodes an intracellular IP3-gated calcium channel involved in calcium signaling [40]. Mutations in this gene have been associated with spinocerebellar ataxia [41] and platelet signaling pathways [42], and *DCP1A* is known to play a role in mRNA decay and also in prematurely terminating protein synthesis [43].

As described in the methods, the JLA approach has the potential to amplify a signal taking into consideration random effects of shared sequence identity (linkage) and the fixed effects of marker genotypes (association), thus maximizing the information in a sample of related individuals [12]. With this approach, we had previously found common SNPs, MAF > 5 %, in *SLC2A9* to be significantly associated with SUA levels in Mexican Americans [12]. Our JLA approach also has the ability to detect rare variants which were primarily from the chromosome 3p26 region showing that rare or low frequency variants are more likely to be identified by linkage rather than association. Of the top 6 significant SNPs, 5 of them had MAF ≤ 5 %. Family-based studies provide the best opportunity to identify these rare variants, with Mendelian transmission from parent to offspring offering a chance to maximize copies of rare variants in the pedigree. This was supported by our analyses when we found that about six families contributed the most to the LOD score (~4.6). When we conducted the linkage analysis removing these families, the LOD score was reduced to zero, whereas, linkage analysis in just these families increased the LOD score to 5.5.

The association between SUA concentrations and variants in the chromosome 3p region has not been reported in any population except a study in an isolated population in Europe. This study reported epistasis between *SLC2A9* and *CNTN4* suggesting a link between SUA levels and autism-related spectrum disorder. Purine metabolism disorders have been reported in autism spectrum disorders [44, 45] particularly hyperuricosuric autism. Adenosine, a precursor of uric acid in purine metabolic pathway, is believed to be neuroprotective and known to promote sleep and reduce seizures [46] indicating its potential as a therapeutic agent for autism. Lack of replication of the associations between SUA and *CNTN4* or *ITPR1* SNPs by other studies is a limitation of the study. However, considering the role of purine metabolic disorders in autism, role for *CNTN4* and *ITPR1* in the regulation of SUA seems plausible and needs to be evaluated further.

To gain further support for the association with the chromosome 3p26 region, we conducted JLA of *cis*-regulated *ITPR1* transcript. Our best associations of these transcript levels were with SNPs in *ITPR1* and *SCN8A* genes. *Cis*-regulated transcripts contain genetic variation within their gene and regulatory regions that affect their abundance [29]. The *SCN8A* gene encodes a protein that is important for neuron hyperexcitability [47] and mutations in this gene are known to cause cerebellar ataxia, which is the similar to that of *ITPR1*. Given the importance of 3p region in neurological disorders and potential role of uric acid as biomarker for these disorders, this region assumes significance.

## Conclusion

Our findings demonstrate the importance of variants in chromosome 3p26 region, particularly SNPs in *ITPR1* and *CNTN4*, in the regulation of SUA concentrations in our cohort. The results of this study are very promising, though further work needs to be performed to validate them.

## Methods

### Study population

The San Antonio Family Heart Study (SAFHS) and the San Antonio Family Diabetes/Gallbladder study (SAFDGS): The recruitment for the SAFHS was initiated in 1991, and recruitment for SAFDGS was conducted between 1998 and 2001. Details of study recruitment and related material have been detailed previously [48–51]. Genome-wide association, joint linkage/association, and transcriptional analyses were performed on 1281 individuals, coming from 120 Mexican American families from these two studies, for whom whole genome-wide SNP data and related phenotype data were available.

### Phenotyping

For both SAFHS and SAFDGS, several metabolic, hemodynamic, anthropometric, and demographic variables were collected using standard procedures [46, 48]. Uric acid was measured in serum by a colorimetric assay using uricase and peroxidase [52]. A description of the measurement techniques is given elsewhere [9, 12, 53]. Uric acid levels were rank-inverse-normalized prior to genetic analysis.

### Transcriptional profiling

The transcriptional profiling in the SAFHS was performed in 1281 individuals. The methodology related to isolation of lymphocytes from whole blood, isolation of total RNA, anti-RNA synthesis, amplification and purification and identification of expressed transcripts is described in detail in Göring et al., 2007 [29].

### SNP genotyping

Genome-wide association (GWAS) analysis was conducted in the SAFHS/SAFDGS using SNP genotypes obtained from the Illumina HumanHap550 BeadChip (Illumina, SanDiego, CA). Our experimental error rate (based on duplicates) was 2 per 100,000 genotypes. The average call rate per individual sample was 97 %. Approximately 1 per 1000 genotypes was blanked due to Mendelian errors. Specific SNPs were removed from analysis if they had call rates *<*95 % (about 4000SNPs) or deviated from Hardy–Weinberg equilibrium at a 5 % false discovery rate (FDR) (12SNPs). Missing genotypes were imputed from pedigree data using MERLIN [54]. SNP genotypes were checked for Mendelian consistency using the program SimWalk2 [55]. The estimates of the allele frequencies and their standard errors were obtained using Sequential Oligogenic Linkage Analysis Routines (SOLAR) [56].

### Heritability analysis

We used a variance components decomposition-based method in SOLAR to estimate heritability of serum uric acid and transcript levels of associated genes. Total phenotypic variance can be partitioned into its genetic and environmental components. The fraction of total phenotypic variance (*V*_*P*_) resulting from additive genetic effects (*V*_*G*_) is called heritability and is denoted as *h*^*2*^ *= V*_*G*_*/V*_*P*_ [56]. All traits were adjusted for age, sex and their interaction effects.

### Joint linkage/Association analysis

We used a joint linkage/association (JLA) approach for each SNP, implemented in SOLAR, that tested each saturated model (including linkage and the fixed effect of the SNP) against a null model in which both effects were constrained to zero. All SNPs in the GWAS panel were mapped not only to their physical location but also to their genetic position, given as the nearest integral centiMorgan, based on public data [12]. JLA may improve detection when multiple causal variants are present, as the summed effects of adjacent variants captured by linkage may amplify the fixed effect of each measured marker. A subsidiary measured genotype association analysis (MGA) [57] tested the additive effect of each SNP genotype conditioned on the genome-wide genetic similarity of the relatives (i.e., a standard GWAS corrected for relatedness). The genome-wide significance threshold *p*-value was set at *p* < 3.1 × 10^−7^ using Bonferroni correction for multiple tests based on the effective number of independent SNPs given linkage disequilibrium (LD) within the sample [58].

## Ethics approval and consent to participate

All participants gave written informed consent. Protocol for both studies (SAFHS and SAFDGS) were approved by the Institutional Review Boards at the University of Texas Health Science Center San Antonio and University of North Carolina at Chapel Hill

## Availability of data and material

Major portion of the data used in this study is deposited in dbGAP (T2D-GENES Project 2: San Antonio Mexican American Family Studies.dbGAP Study Accession: phs000462.v1.p1. http://www.ncbi.nlm.nih.gov/projects/gap/cgi-bin/study.cgi?study_id=phs000462.v1.p1.

Rest of the data is in the process of being deposited. However, we welcome the opportunity to form formal collaborations with investigators who are working in this research area and are interested in utilizing these data.
